# Cerebro-Spinal Meningitis

**Published:** 1878-10

**Authors:** E. F. Starr

**Affiliations:** Nacoochee, Ga.


CEREBRO-SPINAL MENINGITIS.—In the transactions for 1873 of the Medical Association of Georgia will be found a report made by the Editor of this journal, on meningitis. Views of its nature and pathology, with cases and their treatment are there recorded. Observation of the disease had forcibly impressed the author with the belief of its highly inflammatory character, and, in consequence, active and decidedly antiphlogistic treatment was instituted in every case. These cases took large doses of calomel, and whether the circulation and nervous energies were feeble or active, all were bled, and most of them copiously.

The rate of mortality in these cases, compared favorably with those occurring about the same time in the city, and subjected to tonic treatment. I think the good of mankind, and not egotism, prompts me to say, that under quinine and other tonic treatment seventy-five per cent. fell, and not more than twenty per cent. died under general active blood-letting. This opinion is based on the reports made from time to time by physicians of Atlanta who practiced the supporting plan.

The disease has only occasionally been met with here, since that time, and therefore an opportunity has not been afforded to verify the statistics of 1872-3.

We give below the letter of our esteemed correspondent whose views of the pathology and treatment of meningitis has before appeared in this journal. Discussion on this subject *pro* and *con* will be freely admitted into the journal, for the benefit of mankind.

NACOOCHEE, GA., Oct. 25th, 1878.

DR. J. G. WESTMORELAND,

*My Dear Sir:*—In the May No. of your journal, for last year, you published a note addressed to you, by me, upon the subject of meningitis.

This note was induced by some remarks of your own in a previous number upon the same subject; a subject, than which, there are few of greater interest and importance.

I have been hoping since that to see something more from your pen on the same theme, for I believe you intimated then that you would favor your readers with another paper. Recently I have observed somethiug from another source, which I regard as confirmatory of views formerly expressed.

In the department of analytical and bibliographical notices, of the *American Journal of Medical Sciences*, for October 1878, under the head of, the Transactions of the Mississippi Society, the following occurs:

"Dr. E. W. Hughs describes a terrible epidemic of cerebro-spinal meningitis, which prevailed among negroes employed upon the fortifications around Grenada in the fall and winter of 1862 and 1863. Usually without warning, the patients were seized with chill, intense headache, with pain and stiffness at back of neck, followed irregularly by intermittent fever, vertigo, intolerance of light, sound and touch. Delirium and coma were common, with variable affection of the pupils, deafness, irregular and oppressed breathing and many other symptoms of profound poisoning of the nervous centres. Pain in the head and neck, retraction of the head and stiffness of the muscles of the posterior cervical region were the pathognomonic symptoms. * *

The malady was deemed one of depressed vitality, and quinia, opium, iron, and stimulants were given, but all the patients died. Autopsies revealed intense congestion of the membranes and sinuses of the brain. * * *

Antiphlogistic treatment was then tried—venesection, cups, and cold applications, with full doses of calomel, and Dover's powder. From this change of treatment it is claimed that about one-half the cases were saved. Success depended upon the promptness of the first measures."

Of course, in accute meningitis, remedies—treatment—to be efficient, must be prompt. Hençe a plea for venesection; nothing can be so certainly prompt. This when opportunely used will make time for other means. All must admit, that in the above narrative the difference between "all" and "one half" is very striking.

Permit me to say in passing, that I was pleased with your remarks, critical, appended to an article in the September number. In as much as the article found place in its pages, the criticism was timely, appropriate and called for.

I hope the time will not come when the ATLANTA JOURNAL shall be unable to teach a wiser medical philosophy than that contained in the article alluded to.

Yours truly,
E. F. STARR.

